# High prevalence of TROP2 expression identifies EBV-positive gastric cancer as a candidate for TROP2-directed antibody–drug conjugates

**DOI:** 10.1007/s00423-026-04340-8

**Published:** 2026-10-24

**Authors:** Tristan Wagner, Richard Weiten, Su Ir Lyu, Aylin Pamuk, Axel Heidenreich, Christiane J. Bruns, Alexander Quaas, Hakan Alakus, Nikolai Schleussner

**Affiliations:** 1 Department of General, Visceral, Thoracic and Transplant Surgery, University Hospital Cologne, Kerpenertsr. 62, 50937 Cologne, Germany; 2 Present Address: Department of Surgery, Center for Life Sciences, Beth Israel Deaconess Medical Center, Harvard Medical School, 330 Brookline Ave, Boston, MA 02215 USA; 3 Department of Urology, Uro-Oncology, Robot-Assisted, and Specialized Urologic Surgery, University Hospital Cologne, Cologne, Germany; 4 Institute of Pathology, University Hospital Cologne, Cologne, Germany

**Keywords:** TROP2, Antibody-drug conjugate, Epstein-Barr-Virus, Gastric Cancer

## Abstract

**Purpose:**

Trophoblast cell surface antigen 2 (TROP2) is a glycoprotein expressed on epithelial tumor cells and therefore an established target for antibody-drug conjugates (ADCs). Epstein-Barr virus (EBV)-positive gastric cancer is a molecularly distinct subtype characterized by unique immunological features, including frequent responsiveness to immune checkpoint inhibitors (ICIs). Nevertheless, a subset of these patients fails to benefit from ICI therapy, leaving an unmet need for non-immunotherapeutic treatment options. Tumors lacking TROP2, fail to respond to TROP2-directed ADCs. We therefore assessed the prevalence of TROP2 expression in gastric cancer including the EBV-positive subtype, which has not been characterized to date.

**Methods:**

TROP2 expression was evaluated by immunohistochemistry in 525 patients with gastric cancer classified according to TCGA molecular subtypes, representing the largest Caucasian cohort assessed for TROP2 to date.

**Results:**

TROP2 expression differed significantly across molecular subtypes (p < 0.01), with EBV-positive tumors showing the highest median expression (H-score 150). Complete absence of TROP2, the expression pattern associated with treatment failure, was observed in only 3.7% of EBV-positive tumors, compared with 18.8% of chromosomal instability, 25.4% of genomically stable, and 36.2% of microsatellite instability tumors.

**Conclusion:**

Nearly all EBV-positive gastric cancers therefore express TROP2 at levels compatible with ADC targeting. These findings identify TROP2 as a target in EBV-positive gastric cancer and provide a rationale for clinical investigation of TROP2-directed ADCs in this molecularly defined population, particularly as a non-immunotherapeutic option for patients who fail ICI therapy.

**Supplementary Information:**

The online version contains supplementary material available at https://doi.org/10.1007/s00423-026-04340-8.

## Introduction

Gastric cancer ranks as the fifth most common malignancy worldwide and the third leading cause of cancer-related death [1, 2]. For patients with unresectable advanced or recurrent disease, median overall survival remains limited to 13–20 months despite multimodal.

therapy [3, 4, 5]. Molecular profiling by The Cancer Genome Atlas (TCGA) has revealed substantial genomic heterogeneity within gastric adenocarcinoma, identifying four distinct subtypes: chromosomal instability (CIN), microsatellite instability (MSI), genomically stable (GS), and Epstein-Barr virus (EBV)-positive [6]. These molecular distinctions have informed biomarker-directed treatment strategies, including HER2-targeted therapy with trastuzumab [7],t claudin 18.2-targeted therapy with zolbetuximab [5, 8], and immune checkpoint inhibitors, whose benefits is greatest in MSI-high tumors [9] and tumors with high PD-L1 expressions [3, 4, 7, 10, 11].

Nevertheless, objective response rates with current regimens remain modest (40–60%) and median progression-free survival spans only 6.9–10.5 months [3, 12, 13]. These outcomes highlight a persistent unmet need to identify and validate novel therapeutic targets.

EBV-positive gastric cancer constitutes approximately 5–10% of gastric malignancies globally [14]. With nearly one million gastric cancers diagnosed worldwide each year, this amounts to an estimated 80,000 EBV-associated cases annually surpassing EBV-associated Hodgkin lymphoma and making it the most prevalent EBV-associated neoplasm [12, 14]. Clinically, these tumors are characterized by preferential localization to the proximal stomach, a male predominance, a younger age at presentation (< 60 years), and recurrent somatic mutations [13]. At the epigenetic level, EBV-positive gastric cancers exhibit the most extensive DNA hypermethylation patterns observed among solid malignancies [15]. Paradoxically, despite these aggressive molecular features, EBV-positive gastric cancers demonstrate a more favorable prognosis and a significantly lower frequency of lymph node metastasis compared to their EBV-negative counterparts, findings largely attributed to a highly immunogenic tumor microenvironment [8, 13].

Trophoblast cell surface antigen 2 (TROP2) encoded by the tumor-associated calcium signal transducer 2 (*TACSTD2*) has emerged as a promising therapeutic target across epithelial malignancies, including gastric adenocarcinoma [16, 17 ,18, 19]. TROP2 is a 36-kDa type I transmembrane glycoprotein that functions as a calcium signal transducer and modulates multiple oncogenic signaling cascades [20]. In gastric adenocarcinoma, TROP2 overexpression is observed in approximately 56% of cases and serves as an independent adverse prognostic marker for disease recurrence, correlating with decreased disease-free survival, particularly in intestinal-type histology, lymph node-positive disease, and advanced-stage tumors [21].

Despite the established significance of TROP2 as a therapeutic target in the broader cancer population [22, 23, 24, 25] and the distinctive molecular and immunological features of EBV-positive gastric cancer [6, 12, 15], a critical knowledge gap persists regarding TROP2 expression patterns, prognostic implications, and therapeutic relevance specifically within the EBV-positive molecular subtype. Several lines of evidence suggest that TROP2-directed antibody-drug conjugates (ADCs) may be particularly effective in this context. EBV-positive tumors are characterized by dense CD8 + T-cell infiltration, high tumor mutational burden, PD-L1/PD-L2 overexpression, and clinical responsiveness to immune checkpoint blockade [9, 26, 27] features that collectively define an immune-permissive microenvironment. TROP2-targeted ADCs can induce immunogenic cell death (ICD) through the release of damage-associated molecular patterns (DAMPs) [28, 29], a mechanism that may synergize with this pre-existing antitumor immune milieu to augment therapeutic efficacy beyond direct cytotoxicity. Furthermore, the bystander killing effect of TROP2-targeted ADCs may confer an additional advantage in EBV-positive tumors, extending cytotoxic activity to antigen-heterogeneous tumor cell.

populations [30]. Critically, TROP2 expression itself determines ADC efficacy. In esophageal adenocarcinoma, 88% of 598 tumors expressed TROP2 and complete absence of tumoral TROP2 was associated with a lack of response to the TROP2-directed ADC Sacituzumab Govitecan (SG) in vitro and in vivo, leading to advocate TROP2 testing at the protein level before therapy [31]. Consistently, in the phase III ASCENT trial in metastatic triple-negative breast cancer, response rates to SG declined with decreasing TROP2 expression [32]. Establishing the prevalence of TROP2 loss therefore defines the proportion of patients eligible for TROP2-directed ADCs. To address this gap, we investigated the relationship between EBV status and TROP2 expression in gastric adenocarcinoma and validated the expression pattern in the publicly available TCGA cohort and explored their combined prognostic significance.

## Materials and methods

### Study population and design

We conducted a retrospective, single-center analysis of 737 patients with histologically confirmed gastric adenocarcinoma treated between 1997 and 2016 at the University Hospital of Cologne, Germany. Patients were classified into four molecular subtypes according to The Cancer Genome Atlas (TCGA) framework: chromosomal instability (CIN, *n* = 397), genomically stable (GS, *n* = 61), microsatellite instability (MSI, *n* = 47), and Epstein-Barr virus-positive (EBV, *n* = 27). Detailed clinicopathological characteristics of the study cohort are summarized in Tables 1 and 2. The remaining 205 cases (27.8%) could not be assigned to a molecular subtype and were excluded.

**Table 1:** Characteristics of all patients (*n* = 737)

Characteristic	*n* (%)
Median age, years	65
Age group, years	
11–30	5 (0.7)
31–50	103 (14.0)
51–70	369 (50.0)
71–100	260 (35.3)
Sex	
Male	499 (67.7)
Female	238 (32.3)
Tumor site*	
Gastroesophageal junction	267 (36.3)
Proximal stomach	60 (8.2)
Corpus	193 (26.3)
Distal stomach	162 (22.0)
Whole stomach	19 (2.6)
Gastric stump/anastomosis	34 (4.6)
Clinical M category*	
cM0	538 (83.8)
cM1	104 (16.2)
Extent of resection	
Transhiatal extended gastrectomy	300 (40.7)
Total gastrectomy	318 (43.1)
Total gastrectomy + subtotal esophagectomy	9 (1.2)
Subtotal gastrectomy	70 (9.5)
Gastrectomy of remnant stomach	38 (5.2)
Fundus resection	2 (0.3)
Reconstruction*	
Roux-en-Y with pouch	100 (13.8)
Roux-en-Y without pouch	600 (83.0)
Billroth II	9 (1.2)
Colon interposition	10 (1.4)
Other	4 (0.6)
Lymphadenectomy*	
< D2	20 (2.8)
D2	458 (65.2)
> D2	224 (31.9)
Margin status*	
R0	652 (90.6)
R1/R2	68 (9.4)
Clavien-Dindo classification*	
Grade 0	353 (50.8)
Grade I	80 (11.5)
Grade II	100 (14.4)
Grade IIIa	66 (9.5)
Grade IIIb	65 (9.4)
Grade IVa	9 (1.3)
Grade IVb	4 (0.6)
Grade V	18 (2.6)
Length of hospital stay, mean days	18.95
Overall survival	
3-year overall survival, %	54.9
5-year overall survival, %	47.6

*Data were not available for all patients; percentages refer to cases with available data

**Table 2:** Clinicopathological characteristics and TROP2 expression stratified by TCGA molecular subtype

Characteristic	Total	TCGA molecular subtype				*p*-value
	*n* (%)	CIN	GS	MSI-H	EBV+	
No. of patients	532 (100)	397 (100)	61 (100)	47 (100)	27 (100)	
Sex*						0.093
Male	356 (67.8)	270 (69.2)	37 (60.7)	27 (57.4)	22 (81.5)	
Female	169 (32.2)	120 (30.8)	24 (39.3)	20 (42.6)	5 (18.5)	
Age, years*						0.002
< 65	176 (43.3)	124 (41.5)	30 (57.7)	8 (23.5)	14 (66.7)	
≥ 65	230 (56.7)	175 (58.5)	22 (42.3)	26 (76.5)	7 (33.3)	
Perioperative/neoadjuvant therapy*						0.276
No	331 (75.7)	240 (73.8)	39 (76.5)	34 (87.2)	18 (81.8)	
Yes	106 (24.3)	85 (26.2)	12 (23.5)	5 (12.8)	4 (18.2)	
(y)pT category*						0.816
(y)pT1	52 (10.7)	42 (11.7)	4 (6.9)	4 (9.5)	2 (7.4)	
(y)pT2	151 (31.1)	113 (31.5)	15 (25.9)	13 (31.0)	10 (37.0)	
(y)pT3	197 (40.5)	142 (39.6)	28 (48.3)	15 (35.7)	12 (44.4)	
(y)pT4	86 (17.7)	62 (17.3)	11 (19.0)	10 (23.8)	3 (11.1)	
(y)pN category*						0.450
(y)pN0	133 (30.2)	98 (30.1)	11 (21.6)	15 (37.5)	9 (39.1)	
(y)pN1	111 (25.2)	82 (25.2)	14 (27.5)	9 (22.5)	6 (26.1)	
(y)pN2	89 (20.2)	67 (20.6)	8 (15.7)	8 (20.0)	6 (26.1)	
(y)pN3	107 (24.3)	79 (24.2)	18 (35.3)	8 (20.0)	2 (8.7)	
Laurén classification*						< 0.001
Intestinal	123 (40.6)	87 (39.7)	4 (11.1)	22 (75.9)	10 (52.6)	
Diffuse	147 (48.5)	105 (47.9)	31 (86.1)	4 (13.8)	7 (36.8)	
Mixed	33 (10.9)	27 (12.3)	1 (2.8)	3 (10.3)	2 (10.5)	
TROP2 expression						0.019
None (H-score 0)	110 (20.7)	76 (19.1)	16 (26.2)	17 (36.2)	1 (3.7)	
Low (H-score 1-100)	224 (42.1)	173 (43.6)	25 (41.0)	12 (25.5)	14 (51.9)	
High (H-score > 100)	198 (37.2)	148 (37.3)	20 (32.8)	18 (38.3)	12 (44.4)	

*Data were not available for all patients. *CIN* chromosomal instability, *GS* genomically stable, *MSI-H* microsatellite instability-high, *EBV+* Epstein-Barr virus-positive

Histopathological classification was performed in accordance with the 8th edition of the American Joint Committee on Cancer/Union for International Cancer Control (AJCC/UICC) TNM staging system and the 5th edition of the World Health Organization (WHO) Classification of Digestive System Tumors. Tumor stage, histological subtype, grade of differentiation, and lymph node status were abstracted from institutional pathology reports.

### Immunohistochemistry

Membranous TROP2 protein expression was assessed by immunohistochemistry using a BOND-MAX automated staining system (Leica Biosystems, Wetzlar, Germany) in accordance with an accredited institutional staining protocol. Sections of 3–4 μm thickness were cut from formalin-fixed, paraffin-embedded (FFPE) tissue blocks and mounted on positively charged glass slides. Following deparaffinization and heat-induced epitope retrieval, sections were incubated with a monoclonal anti-TROP2 antibody (clone EPR20043; catalog no. ab214488; Abcam, Cambridge, UK) at a 1:1000 dilution for 32 min at 37 °C. Antibody binding was visualized using the Bond Polymer Refine Detection system (Leica Biosystems) with 3,3′-diaminobenzidine (DAB) as the chromogen, followed by hematoxylin counterstaining.

Assay reliability was ensured by inclusion of positive controls (placental trophoblast tissue and urothelial carcinoma tissue with established strong membranous TROP2 expression) and negative controls (omission of the primary antibody) in each staining run to verify sensitivity, specificity, and technical consistency.

Two independent board-certified pathologists (S.I.L. and A.Q.) evaluated membranous TROP2 expression using the H-score method. The H-score was calculated as follows: H-score = (% of cells with weak staining × 1) + (% of cells with moderate staining × 2) + (% of cells with strong staining × 3), yielding a continuous score ranging from 0 to 300. Tumors without any membranous staining (H-score 0) were classified as TROP2-absent. TROP2 expression was subsequently categorized as absent (H-score 0), low (H-score 1-100) and high (H-score 101–300). For survival analyses, TROP2-absent and TROP2-low tumors were combined (TROP2-low). In cases of interobserver discordance, slides were jointly re-evaluated to achieve consensus.

### Molecular subtype classification

EBV status was determined by chromogenic in situ hybridization (CISH) for EBV-encoded small RNAs (EBER) using the INFORM EBER Probe (Ventana Medical Systems, Tucson, AZ, USA) on a BenchMark ULTRA automated staining platform (Ventana Medical Systems). Cases demonstrating nuclear EBER positivity in tumor cells were classified as EBV-positive.

Microsatellite instability (MSI) status was evaluated by immunohistochemistry for the mismatch repair (MMR) proteins MLH1, MSH2, MSH6, and PMS2. Loss of nuclear expression of at least one MMR protein in tumor cells, with retained expression in adjacent non-neoplastic tissue serving as an internal control, was classified as mismatch repair-deficient (dMMR)/MSI.

Cases negative for both EBV and dMMR/MSI were subsequently classified as chromosomal instability (CIN) or genomically stable (GS) subtypes based on previously established molecular profiling data generated at our institution.

### Validation in the TCGA cohort

To validate the subtype-specific expression pattern in an independent cohort, publicly available data of the TCGA stomach adenocarcinoma cohort (TCGA-STAD) was obtained from the TCGA Pan-Cancer Atlas hub of the UCSC Xena browser [33] (https://xenabrowser.net; accessed September 2026). Molecular subtypes were taken from the Pan-Cancer Atlas subtype annotation [34], in which the hypermutated-indel (HM-indel) subtype corresponds to MSI. Overall survival data were obtained from the TCGA Pan-Cancer Clinical Data Resource [34]. TACSTD2 mRNA expression was used as log2-transformed normalized values. Primary tumors with an assigned molecular subtype, available TACSTD2 expression were included (*n* = 360; EBV-positive, *n* = 30). Seven tumors of the hypermutated-SNV subtype were classified as EBV-negative in analyses by EBV status and excluded from comparisons between subtypes (*n* = 353). Expression across subtypes was compared using the Kruskal-Wallis test, followed by unadjusted pairwise Wilcoxon rank-sum tests with EBV-positive tumors as reference. The association between TACSTD2 expression quartiles and EBV status was assessed using Fisher’s exact test. As a sensitivity analysis, odds ratios with 95% confidence intervals for EBV positivity were calculated for four alternative dichotomizations of TACSTD2 expression; these cut points were derived from the cohort and were not pre-specified. Analyses were performed in R (version 4.6.0; R Foundation for Statistical Computing, Vienna, Austria).

### Statistical analysis

Statistical analyses were performed using IBM SPSS Statistics (version 28.0.1.1; IBM Corporation, Armonk, NY, USA) and GraphPad Prism (version 9.4.0; GraphPad Software, San Diego, CA, USA). Differences in TROP2 expression (H-score) among the four TCGA molecular subtypes (CIN, MSI, GS, and EBV) were compared using the nonparametric Kruskal-Wallis test, given the non-normal distribution of H-score values. Post hoc pairwise comparisons were performed using Dunn’s test with Bonferroni correction for multiple testing, where applicable. Categorical expression levels were compared using the Pearson´s chi-square test. Overall survival (OS) was estimated using the Kaplan-Meier method, and between-group differences were assessed using the log-rank (Mantel-Cox) test. Survival analyses were restricted to patients with available follow-up data. Of the 532 molecularly classified patients, 388 had both an evaluable TROP2 H-score and follow-up information and were included in the analysis of overall survival by TROP2 status (low, *n* = 244; high, *n* = 144), and 375 in the subtype-stratified analysis. All reported p-values were two-sided, and statistical significance was defined at a threshold of *p* < 0.05. Due to the small sample size, the survival analysis within the EBV subgroup was considered exploratory.

### Ethical considerations

This study was approved by the Ethics Committee of the Medical Faculty, University of Cologne (approval no. 23-1178) and conducted in accordance with the ethical principles of the Declaration of Helsinki (2013 revision). Given the retrospective design and exclusive use of archived, anonymized tissue specimens, the Ethics Committee granted a waiver of written informed consent.

## Results

### Clinicopathological characteristics

A total of 737 patients with gastric adenocarcinoma were included in the analysis. The median age at diagnosis was 65 years, and 499 patients (67.7%) were male. Tumors were most frequently located at the gastroesophageal junction (36.3%), followed by the gastric corpus (26.3%) and the distal stomach (22.0%). Synchronous distant metastases were present in 16.2% of patients at the time of diagnosis (Table 1).

### Surgical and perioperative outcomes

Total gastrectomy was performed in 43.1% of cases, and transhiatal extended gastrectomy in 40.7% of cases. Alimentary tract reconstruction was accomplished via Roux-en-Y anastomosis in 96.8% of patients. D2 lymphadenectomy was performed in 65.2% and extended lymphadenectomy (> D2) in 31.9% of cases (Table 1). Microscopically margin-negative (R0) resection was achieved in 90.6% of patients. No postoperative complications were recorded in 50.8% of cases. The postoperative mortality rate was 2.6%, and the mean length of hospital stay was 18.95 days. The 3-year and 5-year overall survival rates for the entire cohort were 54.9% and 47.6%, respectively (Table 1). 75.7% of the patients received no neoadjuvant therapy (Table 2).

### Pathological characteristics

Clinicopathological features stratified by molecular subtype are summarized in Table 2. EBV-positive tumors showed a male predominance (81.5%; *p* = 0.093) and a younger age (< 65 years) distribution (66.7%; *p* = 0.002), whereas perioperative therapy (*p* = 0.276) and pathological T and N categories did not differ significantly across subtypes (*p* = 0.816 and *p* = 0.450, respectively) and advanced primary tumors, classified as (y)pT3-T4, accounted for 58.2% of cases and 30.2% of patients were classified as (y)pN0 as well as 24.3% as (y)pN3. Lauren classification differed significantly between subtypes (*p* < 0.001), with EBV-positive tumors being predominantly of intestinal type (52.6%) (Table 2).

### Distribution of TCGA molecular subtypes and membranous TROP2 expression

**Fig. 1:**
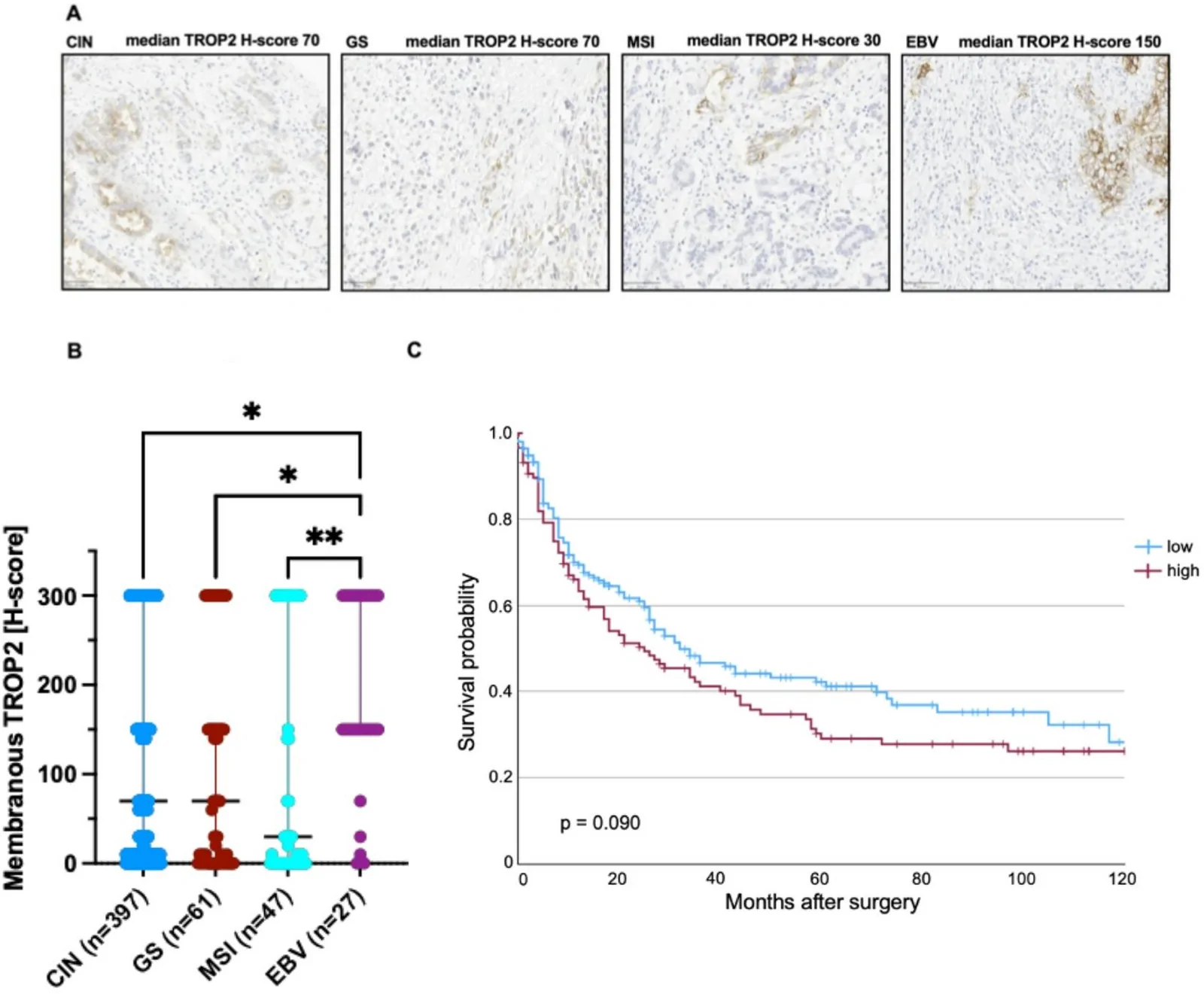
TROP2 expression across TCGA molecular subtypes of gastric adenocarcinoma and association with overall survival. (**A**) Representative immunohistochemistry images demonstrating membranous TROP2 staining patterns across TCGA molecular subtypes of gastric adenocarcinoma (Original magnification, ×200, scale bar, 50 μm). (**B**) Distribution of membranous TROP2 expression (H-score) across molecular subtypes: chromosomal instability (CIN, n = 397), genomically stable (GS, n = 61), microsatellite instability (MSI, n = 47), and Epstein-Barr virus-positive (EBV, n = 27). Each data point represents an individual patient tumor. Horizontal lines indicate median H-scores. EBV-positive tumors demonstrated significantly higher H-scores compared with CIN, GS, and MSI subtypes. (**C**) Kaplan-Meier survival curves comparing overall survival between patients with TROP2-low (blue, n = 244) and TROP2-high (red, n = 144) tumors across the entire cohort. TROP2-high tumors were associated with a shorter median overall survival (25.0 months; 95% CI, 13.3-36.7) than TROP2-low tumors (32.0 months; 95% CI, 21.3-42.7; log-rank p = 0.09). Tick marks indicate censored observations. Statistical significance: *p<0.05, **p<0.01

Molecular classification according to the TCGA framework identified CIN as the predominant subtype (*n* = 397; 74.6%), followed by GS (*n* = 61; 11.5%), MSI-H (*n* = 47; 8.8%), and EBV-positive (*n* = 27; 5.1%) (Table 2). The remaining 205 cases could not be assigned to a molecular subtype and were excluded. Representative immunohistochemical images demonstrating patterns of membranous TROP2 expression across molecular subtypes are shown (Fig. 1A). 

TROP2 protein expression, assessed by H-score, differed significantly across molecular subtypes (*p* < 0.01). EBV-positive tumors displayed the highest median TROP2 expression (median H-score 150; interquartile range [IQR], 150–300), followed by CIN (median H-score 70; IQR, 10–300), GS (median H-score 70; IQR, 0-150), and MSI-H tumors (median H-score 30; IQR, 0-300) (Fig. 1B).

The categorical distribution of membranous TROP2 expression differed significantly across molecular subtypes (*p* = 0.019). Complete absence of TROP2 was rare in EBV-positive tumors (3.7%) compared with the MSI-H (36.2%), GS (26.2%) and CIN (19.1%) subtypes. Consequently, the large majority of EBV-positive tumors expressed TROP2 (low, 51.9%; high, 44.4%), representing the highest proportion of TROP2-expressing cases among all molecular subtypes.

### Validation of TACSTD2 expression in the TCGA cohort

To validate these findings, TACSTD2 mRNA expression was analyzed in the TCGA-STAD cohort (*n* = 360; EBV-positive, *n* = 30). Expression differed significantly across molecular subtypes (Kruskal-Wallis *p* = 7.7 × 10^− 8^). EBV-positive tumors showed the highest median expression, significantly exceeding GS (*p* = 0.00039) and HM-indel/MSI tumors (*p* = 6.2 × 10^− 5^), whereas the difference from CIN tumors did not reach statistical significance (*p* = 0.15) (Supplementary Figure S2A). EBV-positive tumors were enriched among the highest-expressing cases: half of them (15/30) fell into the top expression quartile (Fisher’s exact *p* = 0.018). This corresponded to an odds ratio for EBV positivity of 3.44 (95% CI, 1.49–7.96; *p* = 0.002) for Q4 versus Q1-Q3 and of 4.33 (95% CI, 1.30-18.68; *p* = 0.008) for Q4 versus Q1, whereas associations at lower cutpoints did not reach significance (Supplementary Figure S2B). Thus, the subtype-specific expression pattern observed by immunohistochemistry was reproduced at the mRNA level in an independent cohort.

### Association of TROP2 expression with overall survival in the entire cohort

Kaplan-Meier analysis demonstrated that TROP2 expression in the overall cohort showed a trend toward poorer overall survival, without reaching significance (*p* = 0.090). Patients with TROP2-high tumors (*n* = 144) had a median OS of 25.0 months (95% CI, 13.3–36.7) compared with 32.0 months (95% CI, 21.3–42.7) for those with TROP2-low tumors (*n* = 244). (Fig. 1C).

### Overall survival by TCGA molecular subtype

Survival analysis, stratified by molecular subtype, revealed a significant difference in OS (log-rank *p* = 0.005) (Supplementary Figure S1A). Median OS was 34 months (95% CI, 23.3–44.7) for CIN, 13 months (95% CI, 5.20–20.8) for GS, 59 months (95% CI, 25.7–90.3) for MSI-H tumors and 18 months for EBV-positive tumors (95% CI, 3.9–32.1).

### Exploratory analysis of TROP2 expression and overall survival in EBV-positive tumors

Within the EBV-positive subgroup (*n* = 18), there was no significant difference in the overall survival regarding TROP2-expression (*p* = 0.123). Patients with TROP2-low expression.

(*n* = 9) had a median OS of 4.2 months (95% CI, 1.8–18.2), whereas those with TROP2-high expression (*n* = 9) had a median OS of 48.6 months (95% CI, 0.0-152.2) (Supplementary Figure S1B). Given the small sample size, this analysis is exploratory and should be interpreted with caution.

## Discussion

This study demonstrates that TROP2 expression varies significantly across TCGA molecular subtypes of gastric adenocarcinoma, with EBV-positive tumors exhibiting the highest median expression compared with CIN, GS, and MSI-H subtypes. EBV-positive tumors also demonstrated the most abundant expression of TROP2 with only 3.7% of the patients showing no expression at all. This subtype-specific pattern was reproduced at the mRNA level in the independent TCGA cohort, in which EBV-positive tumors were enriched among the highest TACSTD2-expressing cases. Within the EBV-positive subgroup, overall survival did not differ significantly by TROP2 status.

In the overall cohort, TROP2-high expression showed a trend toward shorter median OS (25.0 vs. 32.0 months), without reaching statistical significance, consistent with the heterogeneous prognostic significance reported for TROP2 in gastric cancer [17, 21, 31, 35]. Taken together, these data suggest that the clinical relevance of TROP2 in gastric cancer lies primarily in its role as a therapeutic target rather than as a prognostic marker.

Mühlmann et al. demonstrated that TROP2 overexpression was an independent predictor of disease recurrence in intestinal-type gastric cancer [21], while a meta-analysis by Zeng et al. confirmed the association between TROP2 overexpression and poor OS across solid tumors (pooled HR, 1.896; 95% CI, 1.60–2.25) [36]. More recently, Kim et al. reported that high TROP2 expression was significantly associated with EBV positivity, intestinal-type histology, and lymph node metastasis, and was an independent predictor of poor survival on multivariate analysis [35]. Our study extends these observations, which were derived from an Asian cohort, to a Western population and quantifies the frequency of complete TROP2 absence across molecular subtypes.

In contrast to the favorable prognosis of EBV-positive gastric cancer reported in the literature, median OS of EBV-positive tumors in our cohort was not longer than that of CIN tumors, which may reflect the small subgroup size and the characteristics of this Western, surgically treated cohort. EBV-positive gastric cancers are characterized by dense CD8 + T-cell infiltration, PD-L1/PD-L2 overexpression, and recurrent PIK3CA mutations, features that collectively define an immune-permissive microenvironment and likely account for the overall favorable prognosis of this subtype [9, 26, 34, 35]. Within the EBV-positive subgroup, overall survival did not differ significantly by TROP2 status. Although TROP2 has been linked to tumor-intrinsic oncogenic signaling, including activation of EGFR/AKT and disruption of desmosomal adhesion via DSG2 downregulation, our exploratory data do not allow conclusions on a prognostic role of TROP2 in this subtype [20, 35, 36, 37]. Notably, CD8 + T-cell infiltration has been reported to exert subtype-specific prognostic effects in gastric cancer. From a therapeutic perspective, the high prevalence of TROP2 expression in EBV-positive gastric cancer (96.3% positivity) positions this subtype as a candidate population for TROP2-directed ADCs. Also, the high prevalence of TROP2 in the overall cohort (approximately 80%) suggests that targeting TROP2 may represent a therapeutic option for gastric cancer patients. TROP2-targeted ADCs, including Sacituzumab Govitecan and Datopotamab deruxtecan, deliver topoisomerase I inhibitor payloads to TROP2-expressing tumor cells and exert additional antitumor activity through the bystander killing effect, whereby released payload diffuses to adjacent antigen-negative cells [18, 37, 38]. Notably, complete absence of TROP2, the expression pattern shown to abrogate SG efficacy in esophageal adenocarcinoma, was found in only 3.7% of EBV-positive tumors, indicating that nearly all patients within this subtype would be eligible for TROP2-directed ADC therapy. This distinguishes EBV-positive tumors from other subtypes: although TROP2 is expressed in the majority of gastric cancers, approximately 20% of CIN tumors, one quarter of GS tumors and more than one third of MSI-H tumors lack TROP2 entirely and would therefore be unlikely to benefit from TROP2-directed ADCs. The clinically relevant difference between subtypes thus lies less in the level of expression than in the proportion of patients who are potentially eligible for treatment. For MSI-H tumors, which appear least suited to TROP2-directed therapy, immune checkpoint inhibitors already represent an established option. The frequency of TROP2 expression observed here is comparable to that reported in esophageal adenocarcinoma, where 88% of 598 tumors were TROP2-positive [31]. This bystander effect may be particularly advantageous in EBV-positive tumors, which can exhibit heterogeneous TROP2 expression. Furthermore, TROP2-targeted ADCs induce immunogenic cell death by releasing damage-associated molecular patterns, including calreticulin, ATP, and HMGB1, thereby promoting dendritic cell maturation and cytotoxic T-lymphocyte activation [28, 29]. This mechanism may synergize with the pre-existing immune-permissive microenvironment of EBV-positive gastric cancer, potentially augmenting therapeutic efficacy beyond direct cytotoxicity. Preclinical evidence supports this rationale: ADC-mediated immunogenic cell death, combined with immune checkpoint blockade, has demonstrated synergistic antitumor responses across multiple tumor models [39]. Given that EBV-positive gastric cancers already exhibit high PD-L1 expression and clinical responsiveness to immune checkpoint inhibitors, combining TROP2-directed ADCs with checkpoint blockade warrants prospective investigation in this molecularly defined population [9, 26, 35].

With regard to immunotherapy, we envision two complementary scenarios. Although early data suggested high response rates to PD-1 blockade in EBV-positive gastric cancer, these observations are based on very small numbers, and EBV status has not been established as a predictive biomarker for immune checkpoint inhibitors [27, 40]. Patients who do not respond to or progress on immune checkpoint inhibitors therefore represent a clinically defined population with limited options. In the near term, TROP2-directed ADCs appear most relevant as a non-immunological treatment option in this setting, in line with the ongoing evaluation of TROP2-directed ADCs in pretreated gastroesophageal adenocarcinoma [41]. In the longer term, the capacity of ADCs to induce immunogenic cell death provides a rationale for combining TROP2-directed ADCs with checkpoint blockade in this immune-permissive subtype, a strategy that has recently improved progression-free survival compared with checkpoint blockade alone in first-line non-small-cell lung cancer [42]. Because nearly all EBV-positive tumors express TROP2, EBV status could be recorded or used as a stratification factor in clinical trials of TROP2-directed ADCs in gastric cancer to test these hypotheses prospectively.

Several limitations of this study merit consideration. First, this is a retrospective, single-center analysis, which introduces potential selection bias and limits generalizability. The molecular subtype distribution may not reflect that of other geographic populations; in particular, the proportion of EBV-positive tumors in our cohort (5.1%) lies at the lower end of the 5% to 18% reported worldwide. Second, the EBV-positive subgroup comprised only 27 patients, limiting statistical power and precluding multivariate adjustment for potential confounders, including stage, treatment modality, and histological subtype. .

Consequently, the survival analysis within the EBV-positive subgroup (*n* = 18 with follow-up; *p* = 0.123) is exploratory and underpowered, and the wide confidence intervals preclude conclusions on a prognostic role of TROP2 in this subtype. This limitation reflects the rarity of EBV-positive gastric cancer, particularly in Western populations; even the largest publicly available dataset, TCGA-STAD, includes only 30 EBV-positive tumors with survival data, in which TACSTD2 expression was likewise not associated with overall survival. Third, the TCGA validation is based on bulk mRNA expression, which does not capture membranous protein localization or complete protein absence and may underestimate epithelial TROP2 expression in lymphocyte-rich EBV-positive tumors. Fourth, the H-score cut-off of 100 used to define TROP2-high expression has not been validated for patient selection for TROP2-directed ADCs. Multicenter or consortium-based cohorts will be required to address these questions.

In conclusion, these findings support the assessment of TROP2 across molecular subtypes, are supported by independent TCGA data at the mRNA level and provide a rationale for investigating TROP2-directed ADCs in EBV-positive gastric cancer, particularly in patients who fail immune checkpoint inhibitors and, in the longer term, in combination with checkpoint blockade. Prospective, multicenter studies with larger EBV-positive cohorts are needed to validate these observations and to determine whether TROP2 expression has predictive value for ADC-based therapies in this molecularly defined population.

## Supplementary information

Below is the link to the electronic supplementary material.

Supplementary Figure 1. Overall survival by TCGA molecular subtype and EBV-positive gastric adenocarcinoma. (**A**) Kaplan-Meier survival curves stratified by TCGA molecular classification: chromosomal instability (CIN, blue, n = 281), genomically stable (GS, red, n = 46), microsatellite instability (MSI, cyan, n = 30), and Epstein-Barr virus-positive (EBV, purple, n = 18); log-rank p = 0.005. Median OS for EBV-positive tumors was 18.0 months (95% CI, 3.9-32.1). Tick marks indicate censored observations. (**B**) Kaplan-Meier survival curves within the EBV-positive subgroup comparing patients with TROP2-low expression (H-score 0-100, blue, n = 9) versus TROP2-high expression (H-score 101-300, red, n = 9; log-rank p = 0.123). Tick marks indicate censored observations

Supplementary Figure 2. TACSTD2 (TROP2) mRNA expression and EBV status in the TCGA-STAD cohort (n = 360). (**A**) TACSTD2 expression [log2(normalized count + 1)] across TCGA molecular subtypes. Molecular subtypes were defined according to the Pan-Cancer Atlas subtype annotation 34, with the hypermutated-indel (HM-indel) subtype corresponding to MSI. (**B**) Odds ratios (95% CI, log scale) for EBV positivity across four alternative dichotomizations of TACSTD2 expression. The lower-expression group served as the reference group for each comparison. Cut points (Q1 ≤ 10.13; median 11.5; Q4 > 12.29) were derived from this cohort and were not pre-specified; the four comparisons represent alternative dichotomizations of the same variable

## Data Availability

The datasets generated and analysed during the current study are not publicly available due to data protection regulations and patient privacy but can be made available by the corresponding author upon reasonable request.
