# Supplementary material for: High prevalence of TROP2 expression identifies EBV-positive gastric cancer as a candidate for TROP2-directed antibody–drug conjugates
Source: Langenbecks Arch Surg. 2026 Oct 24;411(1):290. doi: 10.1007/s00423-026-04340-8 (PMC13695580; doi:10.1007/s00423-026-04340-8)

Supplementary Figure 2.

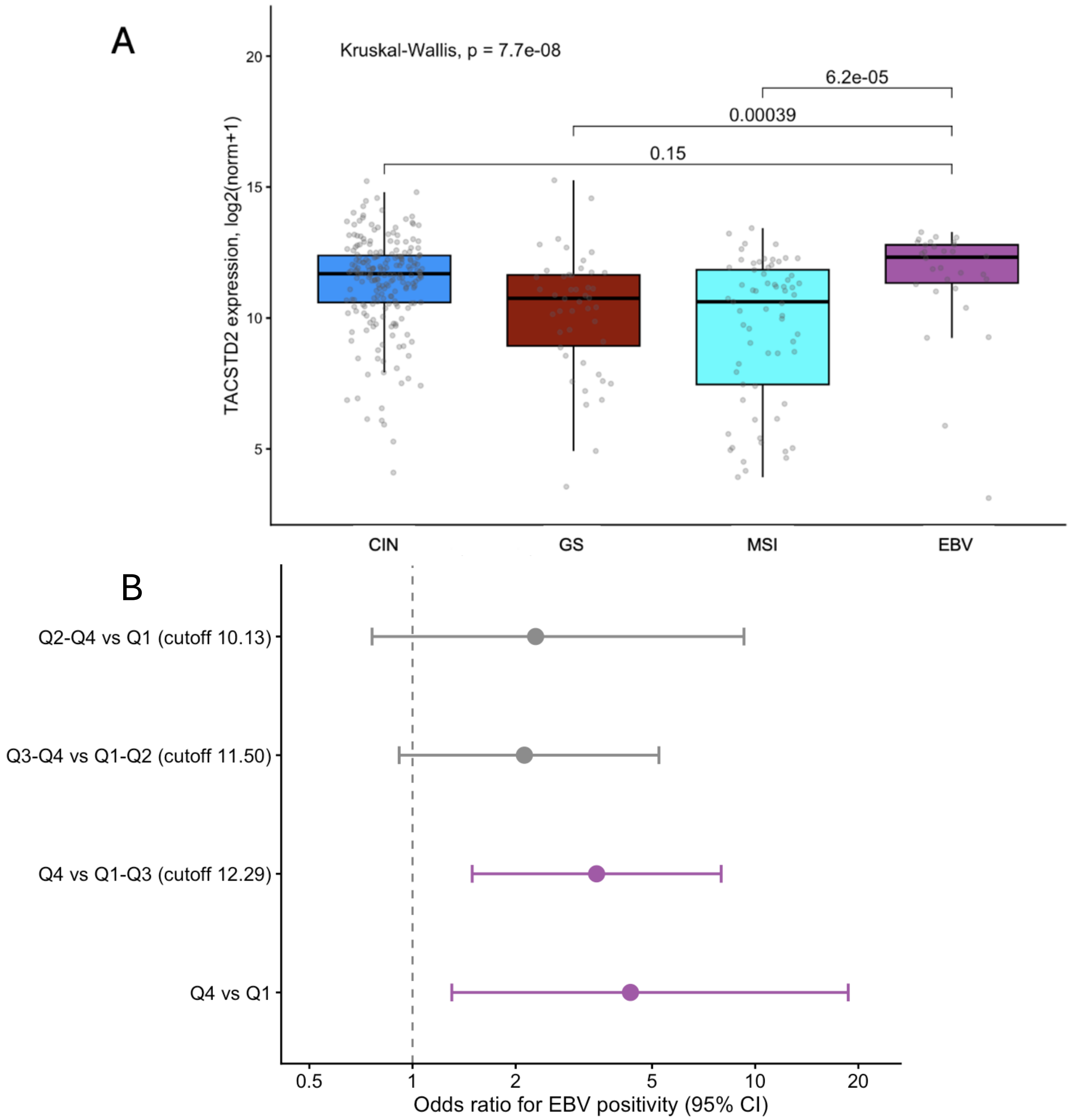

Supplement: Supplementary file 1 [file 423_2026_4340_MOESM2_ESM.pdf]
