# Supplementary material for: High prevalence of TROP2 expression identifies EBV-positive gastric cancer as a candidate for TROP2-directed antibody–drug conjugates
Source: Langenbecks Arch Surg. 2026 Oct 24;411(1):290. doi: 10.1007/s00423-026-04340-8 (PMC13695580; doi:10.1007/s00423-026-04340-8)
Supplement: Supplementary file 2 [file 423_2026_4340_MOESM1_ESM.docx]

**Supplementary Figure 1.**

B

A
